# Practice and consensus-based strategies in diagnosing and managing systemic juvenile idiopathic arthritis in Germany

**DOI:** 10.1186/s12969-018-0224-2

**Published:** 2018-01-22

**Authors:** Claas H. Hinze, Dirk Holzinger, Elke Lainka, Johannes-Peter Haas, Fabian Speth, Tilmann Kallinich, Nikolaus Rieber, Markus Hufnagel, Annette F. Jansson, Christian Hedrich, Hanna Winowski, Thomas Berger, Ivan Foeldvari, Gerd Ganser, Anton Hospach, Hans-Iko Huppertz, Kirsten Mönkemöller, Ulrich Neudorf, Elisabeth Weißbarth-Riedel, Helmut Wittkowski, Gerd Horneff, Dirk Foell, PRO-KIND SJIA project collaborators, Frank Weller, Angelika Thon, Eggert Lilienthal, Thomas Lutz, Prasad T. Oommen, Rainer Berendes, Jens Berrang, Klaus Tenbrock, Christoph Rietschel, Georg Heubner, Rolf-Michael Küster

**Affiliations:** 1 Department of Pediatric Rheumatology and Immunology, University Hospital Münster, Münster, Albert-Schweitzer-Campus 1, Building W30, 48149 Münster, Germany; 2 Department of Pediatric Hematology-Oncology, University of Duisburg-Essen, Essen, Germany; 3 Department of Pediatrics, University Hospital Essen, Essen, Germany; 4 German Center for Pediatric and Adolescent Rheumatology, Garmisch-Partenkirchen, Germany; 5 Department of Pediatric Pulmonology and Immunology, Charité, Berlin, Germany; 6 Department of Pediatrics, StKM GmbH and Technical University Muenchen, Munich, Germany; 7 The Department of Pediatrics I, University of Tuebingen, Tuebingen, Germany; 8 Department of Pediatrics, University Hospital Freiburg, Freiburg, Germany; 9 Division of Pediatric Rheumatology & Immunology, Dr. von Hauner Children’s Hospital, University Hospital Munich, Munich, Germany; 10 Department of Pediatrics, University Hospital Dresden, Dresden, Germany; 11 Department of Women’s & Children’s Health, Institute of Translational Medicine, University of Liverpool, Liverpool, UK; 12 Department of Paediatric Rheumatology, Alder Hey Children’s NHS Foundation Trust Hospital, Liverpool, UK; 13 Department of Pediatric Rheumatology, St. Josef Hospital, Sendenhorst, Germany; 14 Vestian Children’s Hospital, Datteln, Germany; 15 Hamburg Center for Pediatric and Adolescent Rheumatology, Hamburg, Germany; 16 Department of Pediatrics, Olga Hospital, Stuttgart, Germany; 17 Department of Pediatrics, Prof. Hess Children’s Hospital, Bremen, Germany; 18 Department of Pediatrics, Cologne Municipal Hospital, Cologne, Germany; 19 Department of Pediatrics, University Hospital Hamburg, Hamburg, Germany; 20 Department of Pediatrics, Asklepios Hospital, St. Augustin, Germany; 21 University of Cologne, Cologne, Germany

**Keywords:** Systemic juvenile idiopathic arthritis, Diagnosis, Treat-to-target, Glucocorticoids, Biologics, Interleukin-1 blockade, Interleukin-6 blockade

## Abstract

**Background:**

Systemic juvenile idiopathic arthritis (SJIA) is an autoinflammatory disease associated with chronic arthritis. Early diagnosis and effective therapy of SJIA is desirable, so that complications are avoided. The PRO-KIND initiative of the German Society for Pediatric Rheumatology (GKJR) aims to define consensus-based strategies to harmonize diagnostic and therapeutic approaches in Germany.

**Methods:**

We analyzed data on patients diagnosed with SJIA from 3 national registries in Germany. Subsequently, via online surveys and teleconferences among pediatric rheumatologists with a special expertise in the treatment of SJIA, we identified current diagnostic and treatment approaches in Germany. Those were harmonized via the formulation of statements and, supported by findings from a literature search. Finally, an in-person consensus conference using nominal group technique was held to further modify and consent the statements.

**Results:**

Up to 50% of patients diagnosed with SJIA in Germany do not fulfill the International League of Associations for Rheumatology (ILAR) classification criteria, mostly due to the absence of chronic arthritis. Our findings suggest that chronic arthritis is not obligatory for the diagnosis and treatment of SJIA, allowing a diagnosis of probable SJIA. Malignant, infectious and hereditary autoinflammatory diseases should be considered before rendering a diagnosis of probable SJIA. There is substantial variability in the initial treatment of SJIA. Based on registry data, most patients initially receive systemic glucocorticoids, however, increasingly substituted or accompanied by biological agents, i.e. interleukin (IL)-1 and IL-6 blockade (up to 27.2% of patients). We identified preferred initial therapies for probable and definitive SJIA, including step-up patterns and treatment targets for the short-term (resolution of fever, decrease in C-reactive protein by 50% within 7 days), the mid-term (improvement in physician global and active joint count by at least 50% or a JADAS-10 score of maximally 5.4 within 4 weeks) and the long-term (glucocorticoid-free clinically inactive disease within 6 to 12 months), and an explicit treat-to-target strategy.

**Conclusions:**

We developed consensus-based strategies regarding the diagnosis and treatment of probable or definitive SJIA in Germany.

**Electronic supplementary material:**

The online version of this article (10.1186/s12969-018-0224-2) contains supplementary material, which is available to authorized users.

## Background

Systemic juvenile idiopathic arthritis (SJIA) is a rare and serious autoinflammatory disorder characterized by systemic inflammation (hectic quotidian fevers, typical rash, serositis, hepatosplenomegaly, lymphadenopathy, acute-phase reaction) and variably accompanied or followed by chronic arthritis [1–3].

The hypothesis exists that early effective treatment of SJIA during a “window of opportunity” may fundamentally affect its long-term outcome and, specifically, reduce the risk of a chronic articular course [4–6]. Hence, early diagnosis and treatment of SJIA may be essential in order to avoid long-term complications. However, guidance on establishing an early diagnosis of SJIA is limited. The existing International League of Associations for Rheumatology (ILAR) classification criteria for SJIA have been criticized [7]. Classification criteria for the closely related and presumably identical condition of adult-onset Still’s disease (AOSD) exist (Yamaguchi or Fautrel criteria) but have not been formally validated in children [8, 9]. The various classification criteria are summarized in Additional file 1: Table S1.

Historically, SJIA has been effectively treated with glucocorticoids, however, at the cost of substantial adverse effects [10, 11]. Recently advances in treatment have been made via the introduction of biologic drugs targeting interleukin (IL)-1 and IL-6 [12–14]. The outcomes of patients with SJIA have improved markedly due to the availability of these effective antirheumatic therapies [6, 12–19]. Evidence-based guidelines for the treatment of SJIA exist in Germany but are limited in their scope [20]. More recently, treatment recommendations based on evidence and expert opinion for patients with SJIA have been developed by the American College of Rheumatology (ACR) in 2011 and updated in 2013, covering a wide array of clinical scenarios [15, 17]. The North American Childhood Arthritis & Rheumatology Research Association (CARRA) has developed consensus treatment plans based on the usual clinical practice of providers within their group [16]. By experience, treatment of SJIA is highly variable among different practitioners and may often be delayed and/or inadequate; data from inception cohorts and registries indicate that inactive disease is often reached late, for example, beyond the first year of treatment [21, 22]. While there are cross-sectional data on the treatment and outcomes of SJIA in Germany, these data do not allow a precise analysis of treatment steps taken by pediatric rheumatologists in this country [21]. Furthermore, while treat-to-target and tight-control have been central principles in the care of adult patients with rheumatoid arthritis [23], these principles have not been integrated into available recommendations for the treatment of JIA. However, some authors have suggested developing treat-to-target strategies for pediatric rheumatology as well [24, 25].

The PRO-KIND (PROjekte zur Klassifikation, Überwachung und Therapie in der KINDerrheumatologie; projects for the classification, monitoring and therapy in pediatric rheumatology) initiative is a sub-committee of the German Society for Pediatric Rheumatology (GKJR) and aims to define consensus-based strategies to harmonize diagnostic and treatment approaches in Germany. This initiative was started since it was perceived that children with juvenile rheumatic diseases in Germany are currently often treated too late or not with the most up-to-date therapeutic options. Overall, the long-term goal of this project is to improve the quality of care and outcome for patients with SJIA. To meet this challenge, the goals of the PRO-KIND initiative are to foster the use of harmonized standardized diagnostic and therapeutic strategies with defined targets.

## Methods

### PRO-KIND SJIA project group and expert panel

The SJIA project group was initiated in October 2015. Altogether, 11 experienced pediatric rheumatologists participated in several online surveys and five telephone conferences. They collected and analyzed the literature, planned the process and drafted different statements. This included the following key steps: 1) Planning and consensus on aims of the project group, 2) retrieval of real-life patient data from 2 registries in Germany (see study populations below), 3) retrieval of evidence from literature on the following topics: Diagnostic/classification criteria or case definitions, diagnostics or differential diagnosis, therapeutic targets, monitoring, and medications, 4) a survey of diagnostic approaches based on clinical case scenario (in addition to members of the core project group, another 21 pediatric rheumatologists with particular experience with SJIA participated, together forming the expert panel), 5) survey analysis by the project group, and formulation of statements based on the aforementioned steps, and 6) discussion and consensus of all statements in a revised form during a face-to-face consensus conference organized in June 2016. Members of the project group were experienced in the management of patients with SJIA and had managed up to 150 patients with SJIA in the last 5 years (median: 15 patients); 6 out of 11 members of the expert panel had previously pursued research in SJIA. The expert panel was formed by additionally recruiting pediatric rheumatologists from the 13 top referring centers to the Autoinflammatory Disease registry (AID-Net) and all centers participating in the inception cohort of newly diagnosed patients with JIA (ICON-JIA).

### Study populations

Data was retrieved from 2 registries and 1 inception cohort regarding the current practice of diagnosing and treating SJIA in Germany. For this study, all patients who entered the registries with a diagnosis of SJIA were analyzed whether ILAR classification criteria were fulfilled or not.

1. The national pediatric rheumatology database (Kerndokumentation) is a central registry maintained by the German Rheumatism Research Center in Berlin since 1997, with the goal to monitor most patients with pediatric rheumatic diseases longitudinally. Clinical data, including current and past antirheumatic therapies, physician global disease activity, joint examination findings, and pertinent laboratory findings are entered annually. Overall 61 pediatric rheumatology centers or providers participate in this database (30 children hospitals, 20 university hospitals and 11 pediatric rheumatologists in private practice). Data from 2007 until 2013 were available for analysis.
2. ICON-JIA in Germany is a controlled observational cohort study to observe patients with a recent diagnosis of JIA, i.e. within 12 months before enrollment, for at least 10 years, including 11 large German pediatric rheumatology centers. ICON started in 2010 and is collecting various data, every 3 months during the first year and every 6 months thereafter. In addition to 975 patients with JIA, 489 children without JIA were included in the cohort.
3. AID-Net since 2009 is monitoring patients of 42 German pediatric rheumatology centers with various autoinflammatory diseases including SJIA and collects detailed clinical and laboratory data during routine and acute visits.

### Parameters retrieved

The following parameters regarding the diagnosis of SJIA were retrieved for this study from ICON-JIA and the AID-Net: prevalence of arthritis, fever, rash, lymphadenopathy, hepatosplenomegaly, serositis, pharyngitis, leukocyte count, C-reactive protein (CRP) level, erythrocyte sedimentation rate (ESR) and S100 protein levels. Regarding treatment of SJIA, medications used in the first year of therapy were retrieved from the 3 databases, specifically nonsteroidal anti-inflammatory drugs (NSAIDs), systemic glucocorticoids, intraarticular glucocorticoids, methotrexate, anakinra, canakinumab, tocilizumab, adalimumab, etanercept, and cyclosporin A. Furthermore, where available, information on the disease course (monocyclic, polycyclic, chronic) and the attainment of clinically inactive disease was retrieved [26].

### Online survey of current clinical practice

In addition, to better understand current concepts in clinical practice, an online survey depicting 6 different clinical scenarios was developed. It had the goal to reflect a spectrum of clinical findings that may be seen in patients with suspected, probable or definitive SJIA (Additional file 2: Table S2). These clinical scenarios contained information about pertinent history of present illness, family history, travel history, pattern of fever, pattern of rash, physical examination findings (including the presence or absence of arthritis, arthralgia, myalgia, lymphadenopathy, hepatosplenomegaly, serositis) and essential laboratory data (including complete blood count, CRP, sedimentation rate, ferritin, transaminases, LDH, albumin, fibrinogen, triglycerides, prothrombin time, partial thromboplastin time, D-dimers) as well as physician and patient global scores, Questions were then targeted to determine the various diagnostic approaches, terminology used and treatments rendered. The survey was sent out to the 33 members of the expert panel.

### Statistical analysis

Descriptive statistics were used to report the registry data. The various classification criteria and case definition were applied to the AID-Net and ICON datasets. Since there were missing data, the proportion of patients fulfilling the criteria was also calculated by means of extrapolation, defining for each individual criterion that its prevalence over the entire SJIA population is reflected by the prevalence among those patients for whom the criterion was tested. For the extrapolation, it was assumed that the individual criteria were independent from each other.

### Consensus process

Diagnostic and treatment patterns were retrieved from the registry and inception cohort data, online survey and evidence for selected clinical questions was extracted from the literature. Based on these data, statements were developed and judged via an online survey among the 11 experts from the project group; each statement could be accepted without further comment, conditionally accepted (comment provided) or rejected. Following this online survey, the statements were refined and presented at an in-person consensus conference in Münster, Germany, on June 24, 2016. Twenty experts were present during this consensus conference, and the process was guided by a professional moderator and assisted by 3 scientists who were not pediatric rheumatologists and one patient representative/parent. We used nominal group technique for consensus building. For each individual statement, the following procedures were performed: the statement and its background were presented by an individual expert member who had extracted the statement in question. Subsequently, every participant of the consensus conference had 1 min of time available to raise issues with the statement being discussed. These issues were recorded on a flip-chart. Then there was a vote via an electronic voting system (audience response system) to identify the top 3 items needing further discussion, followed by a 15-min open discussion of these items to further improve the statement. Subsequently a final round of anonymous voting took place during which each participant could either accept or reject the respective statement. Consensus was considered to be present if at least 80% of experts supportive a statement. The overall consensus process is outlined in Additional file 3: Figure S1.

### Level and strength of evidence and grades of recommendation

We used the Oxford Centre for Evidence-based Medicine levels of evidence and grades of recommendation to further support the individual evidence for the developed statements [27]. Levels of evidence range from 1 to 5, and grades of recommendation from A to D.

## Results

### Characteristics of patients diagnosed with SJIA in Germany

Only 59.9 and 57.1% patients diagnosed with SJIA in the AID registry and the ICON-JIA cohort, respectively, had arthritis at any time during the follow-up. In both the AID registry and the ICON-JIA cohort, exact data regarding the length and pattern of fever and the duration of arthritis at time of diagnosis were not available. For the purpose of this analysis, it was assumed that, if fever was documented to be present, the fever pattern was typical, and, if any arthritis was documented, that it met the requirement for ILAR classification. Table 1 demonstrates the patient characteristics in detail. For the AID registry, detailed data regarding age at disease onset, duration of disease and presence or absence of arthritis were available.

**Table 1:** Clinical parameters of patients diagnosed with SJIA within the German AID registry and ICON-JIA

	German AID registry (*n* = 207)		ICON-JIA (*n* = 35)	
Parameter	Number of patients in whom parameter is available	Number of patients in whom the parameter is positive (%)	Number of patients in whom parameter is available	Number of patients in whom the parameter is positive (%)
Fever	207	202 (97.6%)	35	35 (100%)
Arthritis	207	124 (59.9%)	35	20 (57.1%)
Arthralgia (but not arthritis)	207	76 (36.7%)	35	2 (5.7%)
Rash	207	149 (72.0%)	35	30 (85.7%)
Serositis	207	38 (18.4%)	35	6 (17.1%)
Lymphadenopathy	207	44 (21.3%)	35	12 (34.3%)
Hepato- and/or splenomegaly	207	48 (23.2%)	35	16 (45.7%)
Pharyngitis	207	7 (3.4%)	N/A	N/A
Leukocytosis (WBC > 10,000/mm^3^)	154	129 (83.8%)	33	21 (63.6%)
Leukocytosis (WBC > 15,000/mm^3^)	154	92 (59.7%)	33	10 (30.3%)
CRP ≥ 30 mg/l	158	125 (79.1%)	33	15 (45.5%)
ESR ≥ 50 mm/h	122	90 (73.8%)	26	12 (46.2%)
Marked systemic inflammation^a^	115	110 (95.7%)	35	19 (54.3%)
Extremely elevated S100 proteins^b^	30	23 (76.7%)	19	5 (26.3%)

*AID* Autoinflammatory disease, *GKJR* Society for Pediatric Rheumatology, *ICON-JIA* Inception cohort of newly diagnosed patients with juvenile idiopathic arthritis, *ILAR* International League of Associations for Rheumatology, *N/A* Data not available, *SJIA* Systemic juvenile idiopathic arthritis, *WBC* White blood cell count

^a^WBC > 15,000/mm^3^, C-reactive protein> 30 mg/l, and/or erythrocyte sedimentation rate > 50 mm/h

^b^Extremely elevated S100A8/A9 (> 10,000 ng/ml) or S100A12 (> 1500 ng/ml) serum level

### Diagnosis of SJIA and testing in different clinical scenarios

An online survey among experts in the diagnosis and treatment of SJIA was circulated among the expert panel. Twenty-eight of 33 addressees replied (85% return rate). The initial question was whether the scenario was representative of SJIA or not, followed by the question regarding the nomenclature in a case like this. Of note, many experts requested additional diagnostic testing, since only 38 to 58% of experts designated a diagnosis of definitive SJIA based on the initially presented data (Additional file 4: Table S3). The following diagnostic tests were considered to be potentially useful (at least 50% consent), in addition to the parameters that had already been presented as part of the case scenario (data given as mean ± standard deviation across all case scenarios): echocardiography 97,8 ± 3,4%, blood cultures 96.8 ± 2.4%, ultrasound of the abdomen 90.4 ± 9.4%, serum S100 proteins 91.4 ± 2.9%, chest X-ray 85.4 ± 3.6%, serum immunoglobulins 79.9 ± 3.7%, ECG 75.1 ± 8.2%, urine studies 75.7 ± 3.3%, autoantibodies 70.5 ± 5.7%, joint ultrasound 69.9 ± 3.6%, peripheral blood smear 68.6 ± 4.4%, serum complement levels 61.9 ± 6.0%, bone marrow aspiration 55.4 ± 9.4%, serum procalcitonin 50.3 ± 6.2%. Other tests were considered to be essential by fewer than 50% of participant (listed in order of descending frequency): rheumatoid factor, soluble IL-2 receptor levels, serum amyloid A, plasma cytokines, lymphocyte subpopulations, lumbar puncture, NK cell degranulation, pulmonary function testing, NK cell function, whole body MRI, MRI of painful joints or PET-CT.

### Current pharmacologic treatment based on registry and cohort data

Data on the treatment and disease course of SJIA was retrieved from the national pediatric rheumatology database, the ICON-JIA cohort and the AID-Net registry (Table 2).

**Table 2:** Treatment patterns and disease courses of new-onset SJIA in Germany

Database	National pediatric rheumatology database (2011–2013)	ICON-JIA (2010–2015)	AID registry (2008–2015)
Number SJIA patients	125	34	251
Initial pharmacologic treatments			
Timeframe assessed	Patients with disease duration of 12 months or less	At time of enrollment (within 12 months of disease onset) and within three months prior	In the first three months of treatment
NSAIDs	55 (44.0%)	29 (85.3%)	145 (57,8%)
Systemic glucocorticoids	75 (60.0%)	33 (97.1%)	178 (70.9%)
Intraarticular glucocorticoids	6 (4.8%)	4 (11.8%)	N/A
Methotrexate	68 (54.4%)	16 (47.1%)	102 (40.6%)
Anakinra	17 (13.6%)	5 (14.7%)	31 (12.4%)
Canakinumab	5 (4.0%)	2 (5.9%)	7 (2.8%)
Tocilizumab	12 (9.6%)	2 (5.9%)	12 (4.8%)
Adalimumab	1 (0.8%)	0 (0%)	0 (0%)
Etanercept	2 (1.6%)	1 (2.9%)	6 (2.4%)
Cyclosporin A	0 (0%)	1 (2.9%)	0 (0%)
Disease course	N/A	Inactive disease: 3 months: 56% 6 months: 64% 9 months: 70% 12 months: 70% 18 months: 68% 24 months: 80%	Sufficient data for 156 pts.: Monocyclic 42 (26.9%) Polycyclic 62 (39.7%) Chronic 52 (33.3%) Among 108 pts. with arthritis: Monocyclic 19 (17.6%) Polycyclic 39 (36.1%) Chronic 50 (46.2%)

*AID* Autoinflammatory diseases, *ICON-JIA* Inception cohort of patients with new-onset juvenile idiopathic arthritis, *N/A* Not available, *NSAIDs* Non-steroidal anti-inflammatory drugs, *SJIA* Systemic juvenile idiopathic arthritis

### Current pharmacologic treatment based on case scenarios

In the aforementioned online survey, in addition to diagnostic procedures, we also queried about treatment decisions. These data are represented in Additional file 5: Table S4. It is apparent that systemic glucocorticoids are a preferred treatment modality for SJIA among experts in Germany since between 58 and 91% experts would suggest using glucocorticoids in the various scenarios. Non-steroidal anti-inflammatory drugs were suggested by 48 to 71% of experts, depending on the scenario, but only as adjunctive therapy. However, biologics (anakinra, canakinumab or tocilizumab) are also preferred frequently as an initial therapy, depending on the scenario by 29 to 50% of experts.

### Development of individual statements in the consensus conference and their evidence

Individual statements regarding the diagnosis and management of SJIA were discussed and consented during the final consensus conference. The agreed upon statements, the consensus level and the level of evidence are shown in Table 3. For statement 1 (case definitions for PRO-KIND strategies), the current SJIA and AOSD classification criteria, and Childhood Arthritis & Rheumatology Research Association (CARRA) case definition were identified and analyzed (see Additional file 1: Table S1). For statement 2 (diagnostics and differential diagnosis), a literature search was initiated regarding various blood biomarkers routinely available in Germany (see Additional file 6: Table S5). Several reports were identified discussing the occurrence of malignancy mimicking SJIA, therefore supporting statement 3A [28–31]. Multiple infections may mimic some or most of the clinical manifestations of SJIA. Since the probability for various infections strongly depends on clinical circumstances, exposures and risk factors, the working group concluded that testing for specific infections was not warranted under all circumstances and that, rather, a reasonable clinical approach regarding testing for infections should be used as outlined in the German guideline in respect to fever of unknown origin [32]. There was insufficient data to represent the utility of various imaging studies in the diagnosis of SJIA and statement 3C is based on expert opinion. Since most patients with SJIA demonstrate an excellent response to glucocorticoid, anti-IL-1 or anti-IL-6 therapy, the lack of such a response should prompt reconsideration of the diagnosis of SJIA (statement 3D) [12–14, 33]. Statement 4 defines the treatment targets. Essentially, the treatment targets should be reached in succession, indicating that the priority in SJIA therapy is control of systemic inflammation followed by overall well-being and control of arthritis. If a treatment goal is not reached, changes in treatment are required. For this purpose, the initial treatment target focuses on the absence of fever and marked reduction in CRP (by at least 50%) and the interim target includes the physician global assessment and the count of joints with active arthritis and/or the juvenile arthritis disease activity score (JADAS)-10 which has been validated for different categories of JIA, including SJIA [34]. Statement 5 addresses the available treatment options for patients with SJIA, indicating strong evidence for the efficacy of using glucocorticoids, IL-1 and IL-6 blockade. Statement 6 addresses the treatment of patients with probable SJIA, including a preference for treatment with high-dose glucocorticoids and/or IL-1 blockade. Statement 7 tackles the treatment of patients with definitive SJIA, also addressing specific issues, such as the use of methotrexate and alternative biologics, including tumor necrosis factor (TNF) blockers and abatacept. Table 5 summarizes our case definitions for patients with probable and definitive SJIA. Of note, our case definition for definitive SJIA is similar to the CARRA case definition, i.e. the presence of any arthritis for any length of time would satisfy the entry criteria. The ILAR criteria, formally requiring 6 weeks of arthritis are felt to be unrealistic in the real world since most patients require treatment much earlier.

**Table 3:** Consensus statements regarding the diagnosis and management of SJIA

Statements	Consensus	Levels of evidence and grades of recommendation^a^
Statement 1 Strategies of the PRO-KIND SJIA project group apply to the following patients with new-onset disease:		
(A) Patients with systemic juvenile idiopathic arthritis according to ILAR categorization	100%	1a, A
(B) Patients with suspected SJIA who do not fulfill the ILAR criterion of arthritis	100%	4, D
Statement 2		
(A) The demonstration of systemic inflammation, i.e. usually elevated C-reactive protein, erythrocyte sedimentation rate, leukocytes and/or ferritin) is essential for diagnosing SJIA at disease onset	100%	1b, A
(B) Measurement of specific autoantibodies may be useful in order to rule out other conditions.	100%	5, D
(C) Measurement of phagocyte-specific S100 proteins may be helpful to differentiate between SJIA and other diseases associated with fever. There is insufficient data in regards to interleukin-18 and procalcitonin for the diagnosis of SJIA.	100%	2b-4, C
Statement 3		
(A) Malignancies are important differential diagnoses for SJIA. If suspected, an extended panel of diagnostic tests, including chest radiography, ultrasound of the abdomen and lymph nodes, bone marrow aspiration, and, if appropriate, biopsy of lymph nodes or other involved organs should be pursued. The indication for bone marrow aspiration should be reviewed critically prior to initiating a glucocorticoid therapy. An elevated LDH, uric acid and cytopenias represent pertinent findings.	100%	5, D
(B) Infections are important differential diagnoses for SJIA. An adapted search for infections should be pursued (see guideline “Fever of unknown origin”).	100%	5, D
(C) Hereditary autoinflammatory syndromes are important differential diagnoses for SJIA. Molecular genetic testing should be pursued if clinical suspicion for a known hereditary autoinflammatory syndrome exists.	91.7%^b^	5, D
(D) There are no data from controlled studies regarding the utility of imaging studies in the diagnosis of SJIA. However, sonography and MRI are important modalities to assess joint and organ manifestations, to differentiate from other conditions and to monitor disease activity.	100%	5, D
(E) Generally, in case of insufficient response to antirheumatic therapies, especially glucocorticoids, interleukin-1 or interleukin-6 blockade, the diagnosis of SJIA has to be critically reconsidered.	100%	5, D
Statement 4		
(A) The overall treatment target is achieving a clinically inactive disease, ideally without glucocorticoids, and, eventually, clinical remission. Clinically inactive disease is aimed for within six to twelve months.	100%	2A
(B) The following interim targets are aimed for: a. Resolution of fever within one week of the start of treatment b. Improvement of CRP by at least 50% within one week of the start of treatment c. Marked improvement of overall disease activity within four weeks of the start of treatment, i.e. improvement of the physician global disease activity by at least 50%, reduction of actively inflamed joints (if present) by at least 50% and/or a JADAS10-Score of maximally 5.4	100%	2A
Statement 5		
(A) NSAIDs and DMARDS: Optionally, NSAIDs may be used for treating SJIA even though no data from randomized placebo-controlled clinical trials are available. The only approved DMARD for treating SJIA is methotrexate.	92%	4B
(B) Biologics: Positive data from clinical trials are available for IL-1 blockade (anakinra and canakinumab), IL-6 blockade (tocilizumab) and, in a limited fashion, TNF-alpha blockade (etanercept).	90%	1A
(C) Glucocorticoids: High-dose systemic glucocorticoids are an effective and proven treatment for SJIA.	100%	1A
(D) Intraarticular glucocorticoids may be used for treating arthritis in patients with SJIA.	91%	4C
Statement 6		
(A) Initial treatment: In patients with probable SJIA, high-dose systemic glucocorticoids may be used, either as i.v. pulse therapy and/or as daily glucocorticoids with subsequent dose reduction. Alternatively, anakinra may be used, even as a monotherapy without glucocorticoids. The use of canakinumab or tocilizumab is currently discussed.	100%	2A
(B) In case of inadequate response (interim targets not reached), i.v. pulse glucocorticoid therapy may be repeated, or an increased dose of anakinra may be considered. In case of initial exclusive glucocorticoid therapy, IL-1 blockade or IL-6 blockade may be introduced. In case of initial anakinra monotherapy, additional treatment with glucocorticoids or changing to another biologic may be considered.	100%	1A/2A
(C) In case of persistent or recurrent signs of systemic disease activity, biologics (IL-1 blockade or IL-6 blockade) may be introduced (especially in case of previous exclusive glucocorticoid therapy or a glucocorticoid-dependent disease course). If biologics were already introduced, a dose increase or a change of the biologic can be considered.	100%	1A/2A
(D) If arthritis should develop in patients with probably SJIA, the respective treatment strategy for patients with definitive SJIA is used.	100%	4B
Statement 7		
(A) In the case of SJIA with arthritis, high-dose systemic glucocorticoids may be used, either as i.v. pulse therapy and/or as daily glucocorticoids with subsequent dose reduction. Optionally, NSAIDs, methotrexate and intraarticular glucocorticoids may be employed.	100%	2A
(B) Alternatively, IL-1 or IL-6 blockade may be applied, possibly in combination with glucocorticoids and/or methotrexate.	100%	1A
(C) In case of insufficient treatment response (see treatment targets), i.v. glucocorticoid pulse therapy may be repeated, or IL-1 or IL-6 blocking agents may be increased in dose (if feasible). In case of initial glucocorticoid therapy, IL-1 or IL- blockade may be initiated. In case of initial biological monotherapy, glucocorticoids may be added (systemically or locally), the biological agent may be changed, or methotrexate may be added.	100%	1A
(D) In case of a predominant polyarticular arthritis and in case of lack of treatment response despite the utilization of the approved biological agents, second-line agents, e.g. TNF blockers (etanercept or adalimumab) or abatacept may be applied. In addition, the use of methotrexate is reasonable and intraarticular glucocorticoids may be applied.	100%	2B
(E) If MAS should develop in the context of SJIA, the corresponding treatment strategies are used.	100%	4C

*CRP* C-reactive protein, *DMARD* Disease-modifying antirheumatic drug, *IL* Interleukin, *ILAR* International league of associations for rheumatology, *MAS* Macrophage activation syndrome, *NSAIDs* Non-steroidal anti-inflammatory drugs, *SJIA* Systemic juvenile idiopathic arthritis

^a^according to the Oxford Centre for Evidence-based Medicine

^b^consensus was determined in a post-consensus meeting survey among 22 experts

### Application of various classification criteria

The different classification criteria, i.e. the ILAR and Yamaguchi criteria and the GKJR case definitions were applied to the individual patient data in the AID registry and the ICON-JIA cohort (Additional file 7: Table S6). The probability of patients within each registry to fulfill the various criteria and case definition was calculated, indicating that the sensitivity of the ILAR classification was rather poor in the AID registry and the ICON-JIA cohort, identifying only 47.8 and 54.3% when applying the criteria de facto, respectively (Additional file 7: Table S6). In contrast, the Yamaguchi classification criteria identified 55.1 and 77.1% of patients correctly, respectively, and the GKJR case definition identified 62.3 and 65.7%, respectively. The sensitivity improved when applying the criteria to patients with laboratory data obtained within the first month after diagnosis in the ICON-JIA cohort and when accounting for missing data by extrapolation (Table 4).

**Table 4:** Consensus approach to the categorization of definitive or probable SJIA

	Definitive SJIA	Probable SJIA
Inclusion criteria		
Typical fever pattern^a^	+++	+++
Arthritis in at least one joint	+++	–
Typical (evanescent erythematous) rash	+	+
Generalized lymphadenopathy	+	+
Hepatomegaly or splenomegaly	+	+
Serositis	+	+
Marked systemic inflammation^b^	–	+++
Extremely elevated S100 proteins (calgranulins)	–	+
Exclusion criteria		
Infection	X	X
Malignancy	X	X
Hereditary autoinflammatory syndrome	X	X
Requirements	All obligatory criteria are fulfilled and at least one minor criterion.	All obligatory and at least two minor criteria are fulfilled.

+++: obligatory criterion; +: minor criterion; X: exclusion criterion

^a^ fever of at least two weeks’ duration that is documented to be daily (“quotidian”) for at least three days

^b^ marked elevation of C-reactive protein, erythrocyte sedimentation rate, leukocytes/granulocytes and/or ferritin

- not specifically addressed in the definition

*SJIA* Systemic juvenile idiopathic arthritis

### Treatment strategies

**Fig. 1:**
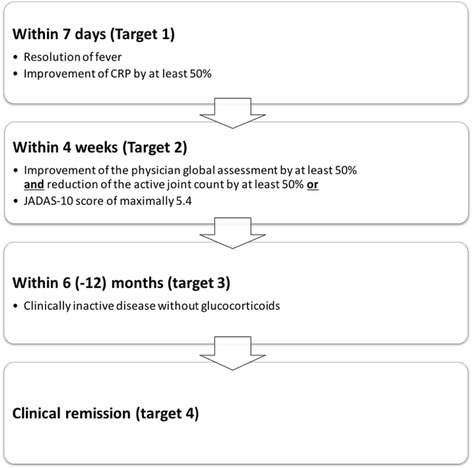
Treatment targets for the treatment of systemic juvenile idiopathic arthritis. Treatments should be reached sequentially. The physician global assessment is scored on a scale of 0 to 10, where 0 represents lack of any disease activity and 10 represents maximal disease activity. The juvenile arthritis disease activity score (JADAS)-10 represents the sum of 4 individual scores, namely the physician global assessment (range 0–10), the patient or parent global assessment (range 0–10), the active joint count (range 0–10), and the normalized erythrocyte sedimentation rate after 1 h ([observed rate - 20]/100, i.e. values up to 20 mm/h are scored as 0 and values equal to or above 120 mm/h are scored as 10) or C-reactive protein (CRP; [observed CRP in mg/l – 10]/100, i.e. values up to 10 mg/l are scored as 0 and values equal to or above 110 mg/l are scored as 10)

**Fig. 2:**
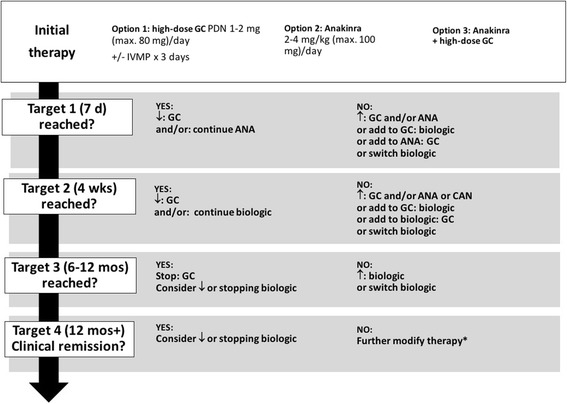
Treat-to-target consensus treatment strategy for the initial therapy of probable systemic juvenile idiopathic arthritis (SJIA). Maximal doses for glucocorticoids: i.v. methylprednisolone pulse therapy (20–30 mg/kg/day [max. 1000 mg/day) for 5 days or prednisolone equivalent 1–2 mg/kg/day (max. 80 mg/day). “Biologic” refers to anakinra, canakinumab or tocilizumab. Maximal doses for biologics: anakinra 8 mg/kg/day (max. 300 mg/day), canakinumab max. 300 mg every 4 weeks, tocilizumab (for body weight > 30 kg) 8 mg/kg (max. 800 mg) i.v. every 2 weeks and (for body weight < 30 kg) 12 mg/kg every 2 weeks. In addition, non-steroidal anti-inflammatory drugs may be used for symptom relief throughout. Combination therapy with biologic agents is discouraged. Abbreviations: ANA, anakinra; CAN, canakinumab; CID, clinical inactive disease; GC, glucocorticoids; IVMP, intravenous methylprednisolone pulse; PDN, prednisone/prednisolone equivalent. *not addressed by these strategies. ↓ = decrease dose or frequency (taper); ↑ = increase dose or frequency

**Fig. 3:**
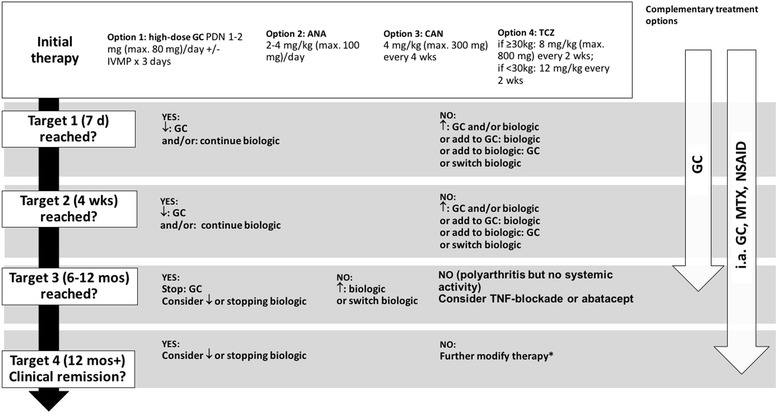
Treat-to-target consensus treatment strategy for definitive SJIA. Maximal doses for glucocorticoids: i.v. methylprednisolone pulse therapy (20–30 mg/kg/day [max. 1000 mg/day) for 5 days or prednisolone equivalent 1–2 mg/kg/day (max. 80 mg/day). “Biologic” refers to anakinra, canakinumab or tocilizumab. Maximal doses for biologics: anakinra 8 mg/kg/day (max. 300 mg/days), canakinumab 8 mg/kg (max. 600 mg) every 4 weeks, tocilizumab (for body weight > 30 kg) 8 mg/kg (max. 800 mg) i.v. every 2 weeks and (for body weight < 30 kg) 12 mg/kg every 2 weeks. In addition, non-steroidal anti-inflammatory drugs may be used for symptom relief throughout. Abbreviations: ANA, anakinra; CAN, canakinumab; CID, clinical inactive disease; GC, glucocorticoids; i.a., intraarticular; IVMP, intravenous methylprednisolone pulse; MTX, methotrexate; NSAID, nonsteroidal anti-inflammatory drug; PDN, prednisone/prednisolone equivalent; TCZ, tocilizumab; TNF, tumor necrosis factor-alpha. ↓ = decrease dose or frequency (taper); ↑ = increase dose or frequency

Treatment strategies for patients with probable/suspected or definitive SJIA were derived from the agreed upon statements. Figure 1 visualizes the treatment targets identified. Figure 2 visualizes the treat-to-target concept and the various ramifications during the first year of treatment of new-onset probable SJIA. The central treatment options for probable SJIA include high-dose systemic glucocorticoids and/or interleukin-1 blockade (anakinra). Figure 3 indicates the options for patients with definitive SJIA. The initial treatment options include high-dose glucocorticoids, anakinra, canakinumab or tocilizumab. In addition, systemic glucocorticoids, intraarticular glucocorticoids, NSAIDs, and/or MTX may be used complementary during the initial treatment. During the course of the disease, for patients who have persistent polyarthritis without systemic inflammation, treatment with tumor necrosis factor-alpha (TNF) blockade or abatacept is an option. Methotrexate is commonly used as an adjunctive therapy in case of polyarthritis and intraarticular glucocorticoid injections may be used in case of persistent arthritis. These treatment strategies represent a harmonization of current SJIA treatment among experts in Germany which is independent of the current approval status of the medications addressed. However, the strategies do not address the management of patients beyond the first year of therapy. Especially in case of refractory SJIA, advice by experts in the management of SJIA should be sought.

## Discussion

We developed practice- and consensus-based statements guiding the management of new-onset SJIA. There is a consensus in Germany that patients with probable SJIA, i.e. patients with a clinical phenotype similar to definitive SJIA but lacking chronic arthritis, may be treated similarly to patients with definitive SJIA. Furthermore, we developed treat-to-target strategies for the management of both probable and definitive SJIA. We emphasize that these strategies are based on the harmonization of current clinical practice and may not represent the optimal way to treat SJIA.

Even though SJIA is currently classified according to the ILAR classification criteria for JIA, its pathogenesis, clinical characteristics and response to therapy are very different from other categories of JIA, and SJIA is tentatively considered an autoinflammatory condition [1, 35]. A presumably identical clinical syndrome has been identified in adults, i.e. AOSD for which classification criteria have also been established, e.g. the Yamaguchi criteria or Fautrel’s criteria [8, 9, 36]. The most important difference between the pediatric and adult criteria is that chronic arthritis is required for the ILAR classification for SJIA, whereas this is not required for the classification of AOSD. In reality, many affected children show all symptoms of SJIA except for arthritis and are nevertheless diagnosed as SJIA [37]. These patients are more resembling of a systemic autoinflammatory disease than of a form of JIA. We support the use of the term “probable SJIA” for these patients; some experts would consider “Still’s disease” more appropriate [7]. It is important to note that important differential diagnoses for patients with suspected SJIA exist, including infectious, malignant or hereditary autoinflammatory diseases. Therefore, before concluding that a patient with merely suspected SJIA has probable SJIA, these differential diagnoses should be considered, and, if necessary, specifically ruled out. Furthermore, our understanding of molecular characteristics of the underlying disease mechanisms is improving [38, 39]. The serum calgranulin proteins S100A8/A9 (calprotectin) and S100A12 are rather sensitive and specific for the detection of active SJIA, and those may be incorporated into the diagnostic approach for SJIA [40–42]. S100 protein testing is currently widely applied in routine clinical practice in Germany. We believe that expert opinion is essential in cases of suspected and probable SJIA.

Our data show that in Germany around 40% of patients receive a diagnosis of SJIA without having chronic arthritis and these numbers exceed those published previously; for example, in another cohort of patients diagnosed with SJIA only around 30% fulfilled the ILAR criteria but 88% had arthritis [37]. We agree with others that patients may be classified as having definitive SJIA even without formally fulfilling the ILAR “entry” criterion of having at least 6 weeks of chronic arthritis [16]. Since the stereotypical disease course is that of a prodromal severe inflammatory phase, variably followed by arthritis, it is unrealistic that patients would manifest 6 weeks of arthritis prior to establishing a diagnosis [2]. Consequently, revised classification criteria for SJIA have been suggested and are in development [7, 43]; our data support such a new classification. Classification criteria are of practical importance in pediatric rheumatology since for the participation in clinical trials classification criteria have to be fulfilled [12, 14]. Therefore, improved classification criteria for SJIA better reflecting the entire spectrum of patients with SJIA are essential [7, 43]. However, classification criteria should not be misused as diagnostic criteria since a delay of diagnosis may lead to delayed treatment and serious complications. Unfortunately, the development of accurate diagnostic criteria is deemed impossible for most rheumatic disorders and, therefore, both the European League against Rheumatism (EULAR) and the American College for Rheumatology (ACR) do not endorse the development of diagnostic criteria [44].

The Yamaguchi criteria and the GKJR case definition may perform better than the ILAR criteria in classifying patients with SJIA. We assume that the GKJR case definition for probable SJIA, requiring laboratory evidence of marked systemic inflammation, may perform better in real life than in the registry data analyzed here. This is based on the notion that the registries did not record inflammatory markers at the time of diagnosis but rather at the time of enrolment, often after treatment had been initiated and inflammatory had already improved.

The optimal treatment for patients with probable SJIA is unclear. Still, based on clinical experience and limited data from published trials in AOSD, it is reasonable to expect that these patients benefit from treatments similar to those for definitive SJIA [7, 33, 45]. In addition, it is an intriguing new concept that the early initiation of an effective therapy may be beneficial by timely rebalancing the immune disturbance that underlies SJIA (within a “window of opportunity”). Therefore, timely treatment may prevent a switch towards the later arthritic phase of the disease [4, 6]. If the concept is correct, prevention of chronic arthritis should be targeted, in addition to treating it.

Effective and proven treatment for SJIA exist and are approved, including glucocorticoids, IL-1 and IL-6 blocking biologicals [12–14]. Some of the treatments discussed in this manuscript relate to non-approved treatment options, for example, anakinra, a recombinant IL-1 receptor antagonist. Even though anakinra is not approved for the treatment of SJIA in Germany, it is frequently used in the initial treatment of SJIA, probably also reflecting current international recommendations and consensus treatment plans [15–17]. We believe that our consensus treatment strategies integrate well with existing international recommendations. It is apparent that most pediatric rheumatologists in Germany and their ways to treat new-onset SJIA are represented in the developed treatment strategies. It is important that the developed strategies do not represent clinical trial protocols, but they rather harmonize variations in typical clinical practice. German physicians should use the consensus treatment plan felt appropriate to use in a given patient and diverge from it whenever this is in the patient’s best interest.

While the existing ACR recommendations and CARRA consensus treatment plans imply a treat-to-target idea, we explicitly embrace a treat-to-target and tight-control approach [16, 17]. Treat-to-target requires the formulation of treatment targets, close monitoring of disease activity and adjustment of treatment if targets are not reached. It has been demonstrated in the management of RA that a treat-to-target strategy improves outcome irrespective of which specific treatments were used [46, 47], essentially indicating that strategy may be more important than individual medications. The ACR recommendations indicate milestones based on the treating physician’s estimation of global disease activity and the active joint count but do not specify milestones earlier than after 2 weeks of treatment changes and beyond 1 month after treatment changes [17].

There is consensus among SJIA experts in Germany that another goal of SJIA treatment is to minimize glucocorticoid exposure and side effects, previously frequently seen in patients with SJIA [11]. The explicit consensus goal is to achieve glucocorticoid-free clinical inactive disease (CID) within 6 to 12 months after initiation of treatment with CID being defined according to the Wallace criteria [26]. Some participants argued for a shorter time frame of 3 months for this goal but there was no consensus for this opinion. The stated goals appear reasonable based on data from clinical trials and outcome data from inception cohorts [21, 22, 48].

Ideally, all patients with SJIA should be offered the opportunity to participate in disease registries allowing the collection of outcome data so that the disease courses and factors affecting the disease course may be better understood.

Furthermore, the consensus treatment strategies we developed should complement existing or future treatment guidelines. Existing treatment guidelines for SJIA are limited by the fact that they are strictly evidence-based; clinically relevant issues, for example, the specific steps when initiating or escalating therapy, are often not addressed [20]. To improve the outcome in patients with SJIA, efforts in harmonizing treatment approaches for SJIA (and other diseases) should be complemented by the collection of outcome data, so that in the long-term the outcome of different treatment strategies may be compared by means of comparative effectiveness research to further optimize treatment strategies. This approach is embodied by the plan-do-study-act improvement cycle used in quality improvement [49].

There are important limitations to our work: These strategies do not address patients with long-standing refractory disease but only patients with new-onset disease. The patient data available from registries were partially incomplete. The data is limited by the fact that temporal resolution is poor for some of the databases. For example, for the national pediatric rheumatology database, data are obtained annually, for ICON-JIA at most quarterly and for the AID-Net irregularly (usually quarterly). For that reason, not all important changes in the clinical parameters and treatment are reflected in the collected data. Therefore, the precise sequence of treatments rendered cannot be deduced from the data available. The available laboratory data in the AID-Net and ICON in most cases do not represent those that occurred during the peak inflammatory phase of the disease onset but rather at the time of enrollment which, in the case of the ICON-JIA cohort, may have been up to 12 months after initial diagnosis. The statements are consensus-based and rarely evidence-based. The outcome of patients with probable SJIA, i.e. an autoinflammatory illness without arthritis, is less well known, especially since they were not included in the landmark clinical trials of SJIA [12–14]. Some of the laboratory parameters discussed are not globally available yet, such as the S100 proteins. The different parameters used in the statistical model of meeting the various classifications are not independent. Furthermore, these consensus statements do not specifically address the management of macrophage activation syndrome (MAS), a life-threatening complication of SJIA. In the future, a separate consensus process including also hematologists and immunologists is planned to develop strategies to manage MAS.

We emphasize that our statements and deductions regarding the diagnosis of probable or definitive SJIA are evidence-informed and consensus-based and may not represent the optimal way to diagnose (and treat) SJIA. However, we believe that harmonization is important in to compare and improve different approaches. The available registries need to be improved so that detailed and relevant information regarding patient outcomes can be extracted. Ideally, once outcome data become available, the strategies can be further optimized based on ongoing circles of quality improvement [50]. Additionally, it is important that the approach considered here is compatible with international recommendations or plans on the diagnosis and treatment of SJIA [15–17].

## Conclusions

In summary, we developed consensus-based statements and strategies on the management of SJIA by harmonization of existing practice patterns among experts in Germany which should aid clinicians in the work-up of patients with suspected JIA and the management of patients with probable and definitive SJIA.

## Additional files

Additional file 1: Tables S1. Analysis of current classification criteria for SJIA and AOSD. (JPEG 47 kb)

Additional file 2: Tables S2. Key components of clinical case scenarios used for the online survey. (DOCX 29 kb)

Additional file 3: Figure S1. Consensus process for the development of statements on the management of systemic juvenile idiopathic arthritis. AID-Net; autoinflammatory disease registry; ICON-JIA, inception cohort for patients with new-onset juvenile idiopathic arthritis; SJIA, systemic juvenile idiopathic arthritis. (DOCX 21 kb)

Additional file 4: Table S3. Results from the online survey on diagnostic considerations and terminology in cases of possible systemic juvenile idiopathic arthritis. (DOCX 22 kb)

Additional file 5: Table S4. Expert opinion on individual management steps in various case scenarios reflecting the spectrum of systemic juvenile idiopathic arthritis. (DOCX 28 kb)

Additional file 6: Table S5. Characteristics of several widely available biomarkers of inflammation in active systemic juvenile idiopathic arthritis. (DOCX 53 kb)

Additional file 7: Table S6. Application of the various classification criteria to patients with systemic juvenile idiopathic arthritis in the German AID registry and the ICON-JIA cohort. (DOCX 22 kb)

## Abbreviations

ACR: American College of Rheumatology
AID: Autoinflammatory disease
AOSD: Adult-onset Still’s disease
CARRA: Childhood Arthritis] Rheumatology Research Association
CID: Clinical inactive disease
CRP: C-reactive protein
ESR: Erythrocyte sedimentation rate
EULAR: European League Against Rheumatism
GKJR: Gesellschaft für Kinder- und Jugendrheumatologie (Society for Pediatric and Adolescent Rheumatology [in Germany])
ICON-JIA: Inception cohort of newly diagnosed patients with JIA
IL: Interleukin
ILAR: International League of Associations for Rheumatology
JADAS: Juvenile arthritis disease activity score
JIA: Juvenile idiopathic arthritis
MTX: Methotrexate
NSAID: Nonsteroidal anti-inflammatory drug
RA: Rheumatoid arthritis
SJIA: Systemic juvenile idiopathic arthritis
