# Supplementary figures and images for: Practice and consensus-based strategies in diagnosing and managing systemic juvenile idiopathic arthritis in Germany
Source: Pediatr Rheumatol Online J. 2018 Jan 22;16:7. doi: 10.1186/s12969-018-0224-2 (PMC5778670; doi:10.1186/s12969-018-0224-2)

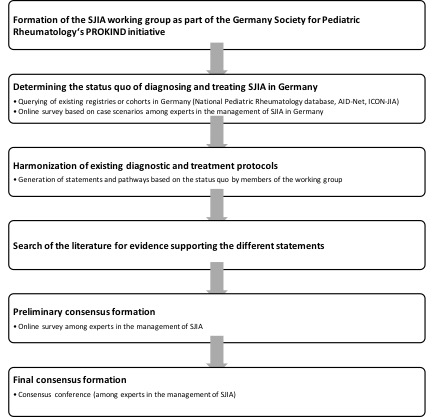

Supplement: Supplementary file 1 — Analysis of current classification criteria for SJIA and AOSD. (JPEG 47 kb) [file 12969_2018_224_MOESM1_ESM.jpg]
