# Supplementary material for: Practice and consensus-based strategies in diagnosing and managing systemic juvenile idiopathic arthritis in Germany
Source: Pediatr Rheumatol Online J. 2018 Jan 22;16:7. doi: 10.1186/s12969-018-0224-2 (PMC5778670; doi:10.1186/s12969-018-0224-2)
Supplement: Supplementary file 2 — Key components of clinical case scenarios used for the online survey. (DOCX 29 kb) [file 12969_2018_224_MOESM2_ESM.docx]

Supplementary Table 1: Analysis of current classification criteria for SJIA and AOSD

| **Type of parameter** | **Parameter** | **Current ILAR classification criteria for SJIA [1]** | **Current case definition for SJIA (CARRA) [2]** | **Yamaguchi classification criteria for AOSD [3]** | **Fautrel classification criteria for AOSD [4]** |
| --- | --- | --- | --- | --- | --- |
| Fever pattern | Fever ≥2 weeks (quotidian ≥3 days) | +++ | +++ |  |  |
|  | Fever (≥39°C) ≥1 wk |  |  | ++ |  |
|  | Spiking Fever (≥39°C) |  |  |  | ++ |
| Joint signs and symptoms | Arthritis of at least 6 weeks duration | +++ |  |  |  |
|  | Any arthritis |  | +++ |  |  |
|  | Arthralgia or arthritis ≥2wks |  |  | ++ |  |
|  | Arthralgia |  |  |  | ++ |
| Cutaneous signs | Evanescent erythematous rash | + | + | ++ |  |
|  | Transient erythema |  |  |  | ++ |
|  | Maculopapular rash |  |  |  | + |
| Other clinical signs | Generalized lymphadenopathy | + | + | + |  |
|  | Hepatomegaly or splenomegaly | + | + | + |  |
|  | Serositis | + | + |  |  |
|  | Sore throat/Pharyngitis |  |  | + | ++ |
| Laboratory data | WBC >10,000/µl (>80% neutrophils) |  |  | ++ |  |
|  | WBC >10,000/µl |  |  |  | + |
|  | >80% neutrophils |  |  |  | ++ |
|  | WBC >15,000/µl with neutrophilia |  |  |  |  |
|  | Abnormal liver function tests |  |  | + |  |
|  | Negative ANA and RF |  |  | + |  |
|  | Glycosylated ferritin ≤20% |  |  |  | ++ |
| Exclusion criteria | Other known conditions | X |  |  |  |
|  | Concomitant infection |  | X | X | X |
|  | Malignancy |  | X | X | X |
|  | Other rheumatic disease |  |  | X | X |
|  | Monogenic AID |  |  |  |  |
|  | ILAR exclusion criterion a)* | X |  |  |  |
|  | ILAR exclusion criterion b)** | X |  |  |  |
|  | ILAR exclusion criterion c)§ | X |  |  |  |
|  | ILAR exclusion criterion d)† | X |  |  |  |
| Requirement for classification | | Two obligatory criteria, ≥1 minor criteria and no exclusion criteria | Two obligatory criteria, ≥1 minor criteria and no exclusion criteria | ≥5 criteria of whom ≥2 must be major criteria and no exclusion criteria | ≥4 major criteria, or 3 major and 2 minor criteria, and no exclusion criteria |
| “+++” indicates an obligatory criterion (sine qua non); “++” indicates a major criterion; “+” indicates a minor criterion; “X” indicates exclusion criteria  1. Petty RE, Southwood TR, Manners P, Baum J, Glass DN, Goldenberg J, He X, Maldonado-Cocco J, Orozco-Alcala J, Prieur AM *et al*: **International League of Associations for Rheumatology classification of juvenile idiopathic arthritis: second revision, Edmonton, 2001**. *The Journal of rheumatology* 2004, **31**(2):390-392.  2. DeWitt EM, Kimura Y, Beukelman T, Nigrovic PA, Onel K, Prahalad S, Schneider R, Stoll ML, Angeles-Han S, Milojevic D *et al*: **Consensus treatment plans for new-onset systemic juvenile idiopathic arthritis**. *Arthritis care & research* 2012, **64**(7):1001-1010.  3. Yamaguchi M, Ohta A, Tsunematsu T, Kasukawa R, Mizushima Y, Kashiwagi H, Kashiwazaki S, Tanimoto K, Matsumoto Y, Ota T *et al*: **Preliminary criteria for classification of adult Still's disease**. *The Journal of rheumatology* 1992, **19**(3):424-430.  4. Fautrel B, Le Moel G, Saint-Marcoux B, Taupin P, Vignes S, Rozenberg S, Koeger AC, Meyer O, Guillevin L, Piette JC *et al*: **Diagnostic value of ferritin and glycosylated ferritin in adult onset Still's disease**. *The Journal of rheumatology* 2001, **28**(2):322-329. | | | | | |
