# Supplementary material for: Practice and consensus-based strategies in diagnosing and managing systemic juvenile idiopathic arthritis in Germany
Source: Pediatr Rheumatol Online J. 2018 Jan 22;16:7. doi: 10.1186/s12969-018-0224-2 (PMC5778670; doi:10.1186/s12969-018-0224-2)
Supplement: Supplementary file 3 — Consensus process for the development of statements on the management of systemic juvenile idiopathic arthritis. AID-Net; autoinflammatory disease registry; ICON-JIA, inception cohort for patients with new-onset juvenile idiopathic arthritis; SJIA, systemic juvenile idiopathic arthritis. (DOCX 21 kb) [file 12969_2018_224_MOESM3_ESM.docx]

Supplementary Table 2: Key components of clinical case scenarios used for the online survey

| **Case** | **Typical systemic findings** | **Arthritis** | **Anemia** | **Acute-phase reaction** | **Disability** | **Comment** |
| --- | --- | --- | --- | --- | --- | --- |
| **1** | ++ | - | ++ | ++ | + | “Probable” SJIA with potential MAS |
| **2** | ++ | + (acute) | + | ++ | ++ | “Definitive” SJIA with potential MAS |
| **3** | ++ | - | + | ++ | ++ | “Probable” SJIA fulfilling Yamaguchi criteria |
| **4** | + | + (acute) | - | ++ | + | “Definitive” SJIA with acute arthritis |
| **5** | + | + | + | + | - | “Definitive” SJIA with chronic arthritis |
| **6** | + | ++ | + | + | + | “Definitive” SJIA with chronic polyarthritis (but atypical course, i.e. first polyarthritis, then fever) |
| MAS, macrophage activation syndrome; SJIA, systemic juvenile idiopathic arthritis  “-“ indicates absence of this finding; “+”, “++” indicate moderate or marked intensity of this finding | | | | | | |
