# Supplementary material for: Practice and consensus-based strategies in diagnosing and managing systemic juvenile idiopathic arthritis in Germany
Source: Pediatr Rheumatol Online J. 2018 Jan 22;16:7. doi: 10.1186/s12969-018-0224-2 (PMC5778670; doi:10.1186/s12969-018-0224-2)
Supplement: Supplementary file 4 — Results from the online survey on diagnostic considerations and terminology in cases of possible systemic juvenile idiopathic arthritis. (DOCX 22 kb) [file 12969_2018_224_MOESM4_ESM.docx]

Supplementary Table 3: Results from the online survey on diagnostic considerations and terminology in cases of possible SJIA

|  | **Case 1** | **Case 2** | **Case 3** | **Case 4** | **Case 5** | **Case 6** |
| --- | --- | --- | --- | --- | --- | --- |
| **Characteristic** | “Probable” SJIA with potential MAS | “Definitive” SJIA with potential MAS | “Probable” SJIA fulfilling Yamaguchi criteria but 14 years old | “Definitive” SJIA with acute arthritis | “Definitive” SJIA with chronic arthritis | “Definitive” SJIA with chronic polyarthritis but atypical course |
| **Initial assessment**  **SJIA ruled out**  **SJIA suspected** | 3.6%  89.3% | 8.0%  92.0% | 4.0%  88.0% | 4.3%  95.7% | 9.1%  86.4% | 14.3%  85.7% |
| **Terminology preferred if further diagnostic studies support the “SJIA” diagnosis**  **Suspected SJIA**  **Probable SJIA**  **SJIA**  **Still syndrome**  **Undifferentiated SAID** | 45.8%  37.5%  4.2%  4.2%  12.5% | 30.4%  26.1%  43.5%  4.3%  - | 38.1%  33.3%  9.5%  4.8%  14.3% | 33.3%  23.8%  38.1%  4.8%  4.8% | 31.6%  10.5%  57.9%  -  5.3% | 23.5%  23.5%  52.9%  -  5.9% |
| SAID, systemic autoinflammatory disease; AOSD; SJIA, systemic juvenile idiopathic arthritis | | | | | | |
