# Supplementary material for: Practice and consensus-based strategies in diagnosing and managing systemic juvenile idiopathic arthritis in Germany
Source: Pediatr Rheumatol Online J. 2018 Jan 22;16:7. doi: 10.1186/s12969-018-0224-2 (PMC5778670; doi:10.1186/s12969-018-0224-2)
Supplement: Supplementary file 5 — Expert opinion on individual management steps in various case scenarios reflecting the spectrum of systemic juvenile idiopathic arthritis. (DOCX 28 kb) [file 12969_2018_224_MOESM5_ESM.docx]

Supplementary Table 4: Expert opinion on individual management steps in various case scenarios reflecting the spectrum of systemic juvenile idiopathic arthritis

|  | **Scenario 1** | **Scenario 2** | **Scenario 3** | **Scenario 4** | **Scenario 5** | **Scenario 6** |
| --- | --- | --- | --- | --- | --- | --- |
| **Core clinical characteristics of this scenario:** |  |  |  |  |  |  |
| Typical systemic findings | ++ | ++ | ++ | + | + | + |
| Arthritis | - | + (acute) | - | + (acute) | + | ++ |
| Arthralgia/myalgia | + | + | + | (+) | (+) | - |
| Global severity of illness | + | ++ | ++ | + | - | + |
| Anemia | ++ | + | + | - | + | + |
| Acute-phase reaction | ++ | ++ | +++ | ++ | + | + |
| Hyperferritinemia  Descriptor | +++  Probable SJIA with possible MAS | +++  SJIA with possible MAS | +  Probable SJIA | +  Acute, classic SJIA | (+)  Chronic oligoarthritis | -  Chronic, severe polyarthritis |
| Survey participants - n | 24 | 23 | 21 | 22 | 19 | 17 |
| **Initial therapy** - n (%)  NSAIDs*  IVMP therapy  Oral PDN  MTX  Anakinra  Canakinumab  Tocilizumab  Cyclosporin A | 13 (54.2%)  20 (83.3%)  10 (41.7%)  4 (16.7%)  10 (41.7%)  1 (4.2%)  1 (4.2%)  0 (0%) | 15 (65.2%)  21 (91.3%)  12 (52.2%)  8 (34.8%)  5 (21.7%)  1 (4.3%)  1 (4.3%)  1 (4.3%) | 10 (47.6%)  13 (61.9%)  8 (38.1%)  3 (14.3%)  9 (42.9%)  0 (0%)  2 (9.5%)  0 (0%) | 13 (59.1%)  17 (77.3%)  8 (36.4)  7 (31.8%)  5 (22.7%)  1 (4.5%)  2 (9.1%)  0 (0%) | 13 (68.4%)  11 (57.9%)  6 (31.6%)  6 (31.6%)  5 (26.3%)  0 (0%)  2 (10.5%)  0 (0%) | 12 (70.6%)  10 (58.8%)  8 (47.1%)  7 (41.2%)  3 (17.6%)  0 (0%)  3 (17.6%)  0 (0%= |
| **When to expect first response to therapy** - median (interquartile range) | 7 (3-14) days | 7 (3-17.5) days | 10.5 (4-14) days | 14 (6-28) days | 10.5 (3-28) days | 14 (3-28) days |
| **Which first treatment response to assess** - n (%) |  |  |  |  |  |  |
| Overall improvement, MD global, improved laboratory values | 23 (95.8%) | 23 (100%) | 20 (95.2%) | 21 (95.5%) | 18 (94.7%) | 16 (94.1%) |
| JADAS below threshold | 3 (12.5%) | 2 (8.7%) | 2 (9.5%) | 2 (9.1%) | 3 (15.8%) | 4 (23.5%) |
| Low disease activity according to ACR | 1 (4.2%) | 0 (0%) | 0 (0%) | 0(0%) | 0 (0%) | 0 (0%) |
| ACR Pediatric 30 response | 1 (4.2%) | 0 (0%) | 0 (0%) | 1 (4.5%) | 0 (0%) | 0 (0%) |
| Clinically inactive disease | 3 (12.5%) | 0 (0%) | 1 (4.8%) | 1 (4.5%) | 1 (5.3%) | 2 (11.8%) |
| **Subsequent therapy if treatment target is not reached** - n (%)  NSAIDs  IVMP therapy  Oral PDN  MTX  Anakinra  Canakinumab  Tocilizumab  Cyclosporin A | 0 (0%)  8 (33.3%)  5 (20.8%)  5 (20.8%)  7 (29.2%)  2 (8.3%)  2 (8.3%)  2 (8.3%) | 3 (14.3%)  7 (33.3%)  5 (23.8%)  7 (33.3%)  5 (23.8%)  1 (4.8%)  7 (33.3%)  1 (4.8%) | 4 (21.1%)  10 (57.9%)  7 (36.8%)  4 (21.1%)  8 (42.1%)  2 (10.5%)  3 (15.8%)  0 (0%) | 4 (18.2%)  1 (50.0%)  8 (36.4%)  5 (22.7%)  9 (40.9%)  2 (9.1%)  6 (27.3%)  0 (0%) | 7 (36.8%)  10 (52.6%)  8 (42.1%)  3 (15.8%)  5 (26.3%)  3 (15.8%)  4 (21.1%)  0 (0%) | 6 (35.3%)  10 (58.8%)  6 (35.3%)  7 (41.2%)  4 (23.5%)  3 (17.6%)  4 (23.5%)  0 (0%) |
| **When to expect clinically inactive disease** - median (interquartile range) | 60 (28-90) days | 90 (28-90) days | 60 (28-90) days | 90 (44-90) days | 90 (28-90) days | 90 (36-90 days) |
| **Subsequent therapy if clinically inactive disease is not reached** - n (%)  NSAIDs*  IVMP therapy  Oral PDN  MTX  Anakinra  Canakinumab  Tocilizumab  Adalimumab  Etanercept  Infliximab  Leflunomide  Abatacept  Rituximab  Cyclosporin A  Azathioprin | 0 (0%)  6 (25%)  4 (16.7%)  4 (16.7%)  2 (8.3%)  7 (29.2%)  14 (60.9%)  0 (0%)  1 (4.2%)  0 (0%)  0 (0%)  0 (0%)  0 (0%)  1 (4.2%)  0 (0%) | 2 (8.7%)  8 (34,8%)  5 (21,7%)  4 (17,4%)  4 (17,4%)  6 (26,1%)  13 (56,5%)  0 ((0,0%)  0 (0,0%)  0 (0,0%)  1 (4,3%)  0 (0,0%)  0 (0,0%)  1 (4,3%)  0 (0,0%) | 2 (9,5%)  7 (33,3%)  4 (19,0%)  2 (9,5%)  1 (4,8%)  6 (28,6%)  9 (42,9%)  0 (0,0%)  0 (0,0%)  0 (0,0%)  1 (4,8%)  0 (0,0%)  2 (9,5%)  1 (4,8%)  0 (0,0%) | 3 (13,6%)  7 (31,8%)  5 (22,7%)  5 (22,7%)  6 (27,3%)  7 (31,8%)  8 (36,4%)  0 (0,0%)  0 (0,0%)  0 (0,0%)  0 (0,0%)  0 (0,0%)  0 (0,0%)  1 (4,5%)  0 (0,0%) | 4 (21,1%)  6 (31,6%)  6 (31,6%)  5 (26,3%)  3 (15,8%)  6 (31,6%)  9 (47,4%)  0 (0,0%)  0 (0,0%)  1 (5,3%)  0 (0,0%)  0 (0,0%)  0 (0,0%)  1 (5,3%)  1 (5,3%) | 5 (29,4%)  5 (29,4%)  6 (35,3%)  4 (23,5%)  3 (17,6%)  4 (23,5%)  8 (47,1%)  1 (5,9%)  0 (0,0%)  0 (0,0%)  1 (5,9%)  1 (5,9%)  1 (5,9%)  1 (5,9%)   1. (0,0%) |
| +++ = severe finding; ++ = strong finding; + = moderate finding; (+) = mild finding; - = not present  NSAIDs were exclusively selected as an adjunctive therapy.  ACR, American College of Rheumatology; IVMP, intravenous methylprednisolone pulse; JADAS, juvenile arthritis disease activity score; MAS, macrophage activation syndrome; MTX, methotrexate; NSAIDs, non-steroidal anti-inflammatory drugs; PDN, prednisone/-olone; SJIA, systemic juvenile idiopathic arthritis | | | | | | |
