# Supplementary material for: Practice and consensus-based strategies in diagnosing and managing systemic juvenile idiopathic arthritis in Germany
Source: Pediatr Rheumatol Online J. 2018 Jan 22;16:7. doi: 10.1186/s12969-018-0224-2 (PMC5778670; doi:10.1186/s12969-018-0224-2)
Supplement: Supplementary file 6 — Characteristics of several widely available biomarkers of inflammation in active systemic juvenile idiopathic arthritis. (DOCX 53 kb) [file 12969_2018_224_MOESM6_ESM.docx]

Supplementary Table 5: Characteristics of several widely available biomarkers of inflammation in active SJIA

| **Parameter** | **Leukocytes** | **C-reactive protein** | **Erythrocyte sedimentation rate** | **Ferritin** | **S100A8/A9** | **S100A12** | **IL-18** | **Procalcitonin** |
| --- | --- | --- | --- | --- | --- | --- | --- | --- |
| **Average level in active SJIA** | 16,100-20,100/µl [1-4] | 24-130 mg/l [1-8] | 38-78 mm/h [1-5] | 935-971 ng/ml [3, 9] | 14920 ng/ml [2] | 7190 ng/ml [4] | 6,240-160,188 pg/ml [10-12] | 0,2 ng/ml |
| **Sensitivity in SJIA** | N/A | 96% [1] | 95% [1] | 70% [1, 9] | 100% (using low threshold) [2] | 100% (using low threshold) [2] | N/D | N/D |
| **Likelihood ratio (control cohorts)** | N/A | 0.99 (Kawasaki disease, infections) [3] | 1.03 (Kawasaki disease, infections) [3] | N/D | 8.0 (systemic infections, NOMID, ALL, AML) [2] | 11.0 (systemic infections), 6.0 (NOMID), N/A since specificity 100% (ALL, AML, MWS) [4] | N/D | N/D |
| **Comparison to control cohorts** | Not significantly different when compared to infections, ALL, AML, KD, FMF or NOMID [2, 3, 5] | Not significantly different when compared to infections, ALL, AML, KD, FMF or NOMID [2, 3, 5] | Not significantly different when compared to infections, ALL, KD, FMF or NOMID [2, 3, 5] | N/D | Significantly elevated when compared to systemic infections, NOMID, ALL, AML | Significantly elevated when compared to systemic infections, NOMID, MWS, ALL, AML; not significantly different from active FMF | Significantly elevated when compared to Kawasaki disease, other categories of JIA [13] | Not significantly different when compared to infections and KD (usually within normal limits) |
| **Comment** | Sensitive but not specific | Highly sensitive but not specific | Highly sensitive but not specific | Moderately sensitive, specificity unclear | Highly sensitive and specific | Highly sensitive and specific | Apparently highly sensitive, specificity unclear | Limited data |
| **Best level of evidence** | 3b | 2b | 3b | 3b | 2b | 2b | 4b | 4b |
| ALL, acute lymphoblastic leukemia; AML, acute myeloid leukemia; FMF, familial mediterranean fever; IL-18, interleukin-18; KD, Kawasaki disease; MWS, Muckle-Wells syndrome; N/A, not applicable; N/D, not determined; NOMID, neonatal-onset multisystem inflammatory disease; SJIA, systemic juvenile idiopathic arthritis  Level of evidence according to the Oxford Centre for Evidence-based Medicine levels of evidence and grades of recommendation [14]  1. Behrens EM, Beukelman T, Gallo L, Spangler J, Rosenkranz M, Arkachaisri T, Ayala R, Groh B, Finkel TH, Cron RQ: **Evaluation of the presentation of systemic onset juvenile rheumatoid arthritis: data from the Pennsylvania Systemic Onset Juvenile Arthritis Registry (PASOJAR)**. *The Journal of rheumatology* 2008, **35**(2):343-348.  2. Frosch M, Ahlmann M, Vogl T, Wittkowski H, Wulffraat N, Foell D, Roth J: **The myeloid-related proteins 8 and 14 complex, a novel ligand of toll-like receptor 4, and interleukin-1beta form a positive feedback mechanism in systemic-onset juvenile idiopathic arthritis**. *Arthritis and rheumatism* 2009, **60**(3):883-891.  3. Shenoi S, Ou JN, Ni C, Macaubas C, Gersuk VH, Wallace CA, Mellins ED, Stevens AM: **Comparison of biomarkers for systemic juvenile idiopathic arthritis**. *Pediatric research* 2015, **78**(5):554-559.  4. Wittkowski H, Frosch M, Wulffraat N, Goldbach-Mansky R, Kallinich T, Kuemmerle-Deschner J, Fruhwald MC, Dassmann S, Pham TH, Roth J *et al*: **S100A12 is a novel molecular marker differentiating systemic-onset juvenile idiopathic arthritis from other causes of fever of unknown origin**. *Arthritis and rheumatism* 2008, **58**(12):3924-3931.  5. Foell D, Wittkowski H, Hammerschmidt I, Wulffraat N, Schmeling H, Frosch M, Horneff G, Kuis W, Sorg C, Roth J: **Monitoring neutrophil activation in juvenile rheumatoid arthritis by S100A12 serum concentrations**. *Arthritis and rheumatism* 2004, **50**(4):1286-1295.  6. Gwyther M, Schwarz H, Howard A, Ansell BM: **C-reactive protein in juvenile chronic arthritis: an indicator of disease activity and possibly amyloidosis**. *Annals of the rheumatic diseases* 1982, **41**(3):259-262.  7. Lotito AP, Campa A, Silva CA, Kiss MH, Mello SB: **Interleukin 18 as a marker of disease activity and severity in patients with juvenile idiopathic arthritis**. *The Journal of rheumatology* 2007, **34**(4):823-830.  8. Rooney M, David J, Symons J, Di Giovine F, Varsani H, Woo P: **Inflammatory cytokine responses in juvenile chronic arthritis**. *British journal of rheumatology* 1995, **34**(5):454-460.  9. Pelkonen P, Swanljung K, Siimes MA: **Ferritinemia as an indicator of systemic disease activity in children with systemic juvenile rheumatoid arthritis**. *Acta paediatrica Scandinavica* 1986, **75**(1):64-68.  10. Maeno N, Takei S, Nomura Y, Imanaka H, Hokonohara M, Miyata K: **Highly elevated serum levels of interleukin-18 in systemic juvenile idiopathic arthritis but not in other juvenile idiopathic arthritis subtypes or in Kawasaki disease: comment on the article by Kawashima et al**. *Arthritis and rheumatism* 2002, **46**(9):2539-2541; author reply 2541-2532.  11. Jelusic M, Lukic IK, Tambic-Bukovac L, Dubravcic K, Malcic I, Rudan I, Batinic D: **Interleukin-18 as a mediator of systemic juvenile idiopathic arthritis**. *Clinical rheumatology* 2007, **26**(8):1332-1334.  12. Shimizu M, Nakagishi Y, Yachie A: **Distinct subsets of patients with systemic juvenile idiopathic arthritis based on their cytokine profiles**. *Cytokine* 2013, **61**(2):345-348.  13. Takahara T, Shimizu M, Nakagishi Y, Kinjo N, Yachie A: **Serum IL-18 as a potential specific marker for differentiating systemic juvenile idiopathic arthritis from incomplete Kawasaki disease**. *Rheumatology international* 2015, **35**(1):81-84.  14. **Oxford Centre for Evidence-based Medicine – Levels of Evidence (March 2009)** [<http://www.cebm.net/oxford-centre-evidence-based-medicine-levels-evidence-march-2009/>] | | | | | | | | |
