# Supplementary material for: Practice and consensus-based strategies in diagnosing and managing systemic juvenile idiopathic arthritis in Germany
Source: Pediatr Rheumatol Online J. 2018 Jan 22;16:7. doi: 10.1186/s12969-018-0224-2 (PMC5778670; doi:10.1186/s12969-018-0224-2)
Supplement: Supplementary file 7 — Application of the various classification criteria to patients with systemic juvenile idiopathic arthritis in the German AID registry and the ICON-JIA cohort. (DOCX 22 kb) [file 12969_2018_224_MOESM7_ESM.docx]

Supplementary Table 6: Application of the various classification criteria to patients with SJIA in the German AID registry and the ICON-JIA cohort

|  | **German AID registry (n=207)** | **ICON-JIA (n=35)** | **ICON-JIA enrolled within first month (n=10)** |
| --- | --- | --- | --- |
| **Parameter** | **Number of patients in whom the criteria are met (%)** | **Number of patients in whom the criteria are met (%)** | **Number of patients in whom the criteria are met (%)** |
| **ILAR classification criteria** | 99 (47.8%) | 19 (54.3%) | 6 (60%) |
| **Yamaguchi classification criteria** | 114 (55.1%) | 27 (77.1%) | 9 (90%) |
| **GKJR case definition for probable SJIA‡** | 51 (24.6%) | 11 (31.4%) | 5 (50%) |
| **GKJR case definition for either probable or definitive SJIA** | 129 (62.3%) | 23 (65.7%) | 9 (90%) |
|  | **% of patients meeting criteria via extrapolation*** | **% of patients meeting criteria via extrapolation*** | **% of patients meeting criteria via extrapolation*** |
| **ILAR classification criteria** | 50.4% | 54.7% | N/A |
| **Yamaguchi classification criteria** | 69.3% | 82.1% | N/A |
| **GKJR case definition for probable SJIA‡** | 71.9% | 57.7% | N/A |
| **GKJR case definition for either probable or definitive SJIA** | 78.3% | 68.0% | N/A |
| AID, autoinflammatory disease; GKJR; Society for Pediatric Rheumatology; ICON-JIA, Inception cohort of newly diagnosed patients with juvenile idiopathic arthritis; ILAR, International League of Associations for Rheumatology; N/A, not applicable; SJIA, systemic juvenile idiopathic arthritis; WBC, white blood cell count  *based on frequency of individual parameters in the registry, assuming independent variables  ‡Typical fever and marked systemic inflammation, and at least two of the following: typical rash, lymphadenopathy, hepatosplenomegaly, serositis, extremely elevated serum S100 protein | | | |
